# A novel heuristic target-dependent neural architecture search method with small samples

**DOI:** 10.3389/fpls.2022.897883

**Published:** 2022-11-07

**Authors:** Leiyang Fu, Shaowen Li, Yuan Rao, Jinxin Liang, Jie Teng, Quanling He

**Affiliations:** ^1^ School of Information and Computer Science, Anhui Agricultural University, Hefei, Anhui, China; ^2^ Department of Science and Technology, Anhui Provincial Key Laboratory of Smart Agricultural Technology and Equipment, Hefei, Anhui, China

**Keywords:** crop classification, target-dependent, neural architecture search, small samples, Bayesian optimization

## Abstract

It is well known that crop classification is essential for genetic resources and phenotype development. Compared with traditional methods, convolutional neural networks can be utilized to identify features automatically. Nevertheless, crops and scenarios are quite complex, which makes it challenging to develop a universal classification method. Furthermore, manual design demands professional knowledge and is time-consuming and labor-intensive. In contrast, auto-search can create network architectures when faced with new species. Using rapeseed images for experiments, we collected eight types to build datasets (rapeseed dataset (RSDS)). In addition, we proposed a novel target-dependent search method based on VGGNet (target-dependent neural architecture search (TD-NAS)). The result shows that test accuracy does not differ significantly between small and large samples. Therefore, the influence of the dataset size on generalization is limited. Moreover, we used two additional open datasets (Pl@ntNet and ICL-Leaf) to test and prove the effectiveness of our method due to three notable features: (a) small sample sizes, (b) stable generalization, and (c) free of unpromising detections.

## Introduction

Image classification can distinguish objects by color, texture, shape, and spatial relationship. It uses computers to analyze images and classify each pixel or region into several categories without human interpretation (Wang and Wang, 2019). The following are two agricultural scenarios. (a) Genetic resources: artificial recognition is time-consuming and near-impossible. Automatic species identification is significant for taxonomy. Purohit et al. (2016) studied a machine-learning method using leaf characteristics to recognize species. In recent years, deep learning, which can automatically extract features from original data, has dramatically improved classification performance (Barré et al., 2017; Lee and Chang, 2017; Pawara et al., 2017). Some studies propose associating machine learning with neural networks (Fu et al., 2016; Li et al., 2020). (b) Phenotype development: a phenotype is a characteristic or combination of an organism influenced by the genotype and by the environment. Usually, plants grow in a highly variable environment. More accurate and robust algorithms are needed to deal with complex backgrounds and quantify phenotypic characteristics. They can distinguish different components or even instances. Li et al. (2020) thoroughly reviewed phenotyping technologies and used machine vision to measure plant stress.

There exist several machine-learning methods such as support vector machines (SVMs) (Rumpf et al., 2010), k-nearest neighbor (KNN) (Rahaman et al., 2019), random forest (RF) (Mohana et al., 2021), and scale-invariant feature transform (SIFT) (Lowe, 2004). However, they have some shortcomings: (a) Classifiers are simple, and the effect of recognition is underperforming. (b) Manual design requires professional knowledge, so it is hard for researchers. Deep learning has rapidly developed in recent years because it can automatically extract features (Lecun et al., 2015) and has achieved excellent performance in vision tasks (Amara et al., 2017). Nevertheless, due to a large number of parameters, designing a good neural network is still a hard task (Suganuma et al., 2017): (a) People made models manually in the early days, which required a lot of professional knowledge (Sun et al., 2020). (b) Neural networks are problem-oriented, whereas manually designed architectures are not. Two main factors affect the performance of neural networks: hyperparameters and training parameters. Training parameters can be learned in the training stage. However, hyperparameters must be set before training. Usually, hyperparameters determine the structure of a network, such as the number and type of layers, kinds of nodes, etc. We hope to find the best hyperparameters for a given dataset in a reasonable time. This process is called hyperparametric optimization. Researchers proved that a gradient descent algorithm is significantly effective for calculating training parameters (Rumelhart et al., 1986). In contrast, there are no explicit strategies for optimizing hyperparameters (Liu et al., 2020).

Most neural networks can be divided into three types: (a) professional knowledge is required for manual design, such as VGG (Ferentinos, 2018) and ResNet (He et al., 2016); (b) semi-automatic design methods like genetic neural networks (Xie and Yuille, 2017), hierarchical evolution (Liu et al., 2017), and others; and (c) fully automatic design, such as when Google introduces the neural architecture search (NAS) concept (Zoph and Le, 2016), which has received considerable attention (Baker et al., 2016; Lu et al., 2018). NAS can search for the best hyperparameters to perform better than manual design. In addition, NAS can reduce trials and errors remarkably. Although NAS is attractive, it still lacks interpretability. Furthermore, model training and verification are costly, and early stopping is meaningful for NAS (Baker et al., 2017; Awad et al., 2021).

This paper studies the hyperparameter optimization of deep learning, and its organization is as follows: (a) Firstly, we examine pertinent technologies. (b) Secondly, the proposed method, as well as the underlying principles, is explained. (c) We then carry out experiments and discuss the results. (d) Finally, conclusions and future research directions are provided.

## Related works

### Deep learning and convolutional neural networks

Deep learning is a branch of machine learning (Deng and Yu, 2014). It is an automatic feature selection strategy based on neural networks. It can combine low-level features to form abstract high-level features without manual selection. Compared with traditional image recognition and target detection methods, the accuracy and generalization are improved. At present, the main types of neural networks are multilayer perceptrons (MLPs), convolutional neural networks (CNNs), and recurrent neural networks (RNNs), among which CNNs are the most widely used method in classification. Generally, CNNs comprise convolution layers, pooling layers, and fully connected layers. A convolution layer uses correlation information to extract features. A pooling layer (mean pooling or max pooling) compresses the amount of data and parameters, reduces overfitting, and keeps the model invariant to translation, rotation, and scaling. Each neuron (also named a node) in a fully connected layer connects with the previous neurons. Therefore, the multidimensional features are integrated and transformed into several dimensions for classification or detection purposes.

Typical CNN models include AlexNet (Picon et al., 2019), VGGNet, GoogLeNet (Liu et al., 2017), ResNet, MobileNet (Howard et al., 2017), etc. AlexNet is the champion network of the ILSVRC-2012, and it includes five convolution layers and three fully connected layers. According to Bengio et al. (2013), deeper CNNs can extract more representative features. Later, researchers found that blindly increasing the number of layers would slow network convergence (Glorot and Bengio, 2010). Microsoft proposed RESNET with residual blocks and fast connections, which made it possible to build a deeper network (Szegedy et al., 2016). Google proposed MobileNet for mobile and embedded vision applications.

The rapid development of deep learning is inseparable from the extensive use of GPUs. Implementations of CNNs mostly require GPUs to provide computing support. CNN processes roughly include (a) data preparation and preprocessing; (b) model development, training, and testing; and (c) model deployment. Usually, a dataset can be divided into training, verification, and test sets, with ratios of 7:2:1, 8:1:1, and 6:2:2. Training sets are for learning parameters; verification sets are for optimizing and adjusting hyperparameters; and test sets evaluate performance and generalization. Some public datasets exist, such as PlantVillage (Sm et al., 2020), Kaggle (Ad et al), etc. It is worth noting that many researchers collect their own (Lin et al., 2018; Chen et al., 2020).

### Data augmentation

The size and diversity of datasets are essential factors affecting the classification effect of CNNs. Data augmentation can expand the number of images, including moving, flipping, zooming, etc. Deep learning can learn features from images regardless of their positions. Therefore, we can expand datasets through augmentation to avoid overfitting. For example, Perez et al. (Perez and Wang, 2017) developed a new way to use generative adversarial networks (GANs) to make images in different styles.

### Neural architecture search

Grid search (GS) is a simple method to find the optimal parameters. However, an exhaustive search may consume time due to the enormous hyperparameter space. Random search (RS) (Andonie and Florea, 2020) explores randomly in the hyperparameter space and improves the performance, but the result may be worse sometimes. For example, the result is not stable. Until now, researchers have proposed many NAS methods: (a) NAS methods based on reinforcement learning (RL); (b) NAS methods based on model optimization; and (c) other improved NAS methods.

#### (a) NAS methods based on reinforcement learning

Researchers designed a controller to generate strings representing the structures of CNNs, trained each CNN model, and used verification set accuracy as a reward. They optimized the hyperparameters of DCNNs using a novel MARL-based approach (multiagent reinforcement learning) (Iranfar et al., 2022). They then created a multiobjective reward function and applied it to reinforcement learning in order to find the best network with the least latency (Tran and Bae, 2021).

#### (b) NAS methods based on model optimization

To improve the performance of neural architecture search, researchers proposed ENAs based on evolutionary computing (EC) (Thomas et al) to design CNN architectures. EC is a population-based technology to obtain an optimal global solution. There are some EC-based technologies, such as genetic algorithms (GAs) (Ching-Shih. Deb et al., 2002), particle swarm optimization (PSO) (Kennedy and Eberhart, 1995), and artificial ant colony algorithms (Dorigo et al., 2006). Researchers proposed a two-stage evolutionary search with transfer learning (EvoNAS-TL) (Wen et al., 2021). Also, EPSOCNN, which stands for efficient particle swarm optimization, is suggested as a way to improve CNN architectures (Wang et al., 2020).

#### (c) Other improved NAS methods

To limit the search space, Yu et al. (2021) and Sun et al. (2019) proposed block-based methods. However, the results are insufficient and unstable due to the lack of theoretical support. Hu et al. (2021) proposed a new performance estimation metric named random-weight evaluation (RWE) to quantify the quality of CNNs. Lu et al. (2018) proposed NSGANet, an evolutionary algorithm that combines prior knowledge from handcrafted architectures with an exploration comprising crossover and mutation. Some software packages provide search functions, such as pyGPGO and Optunity (Bergstra et al., 2011), Hyperopt–Sklearn (Bergstra et al., 2015), etc.

Many CNN models are challenging to apply on mobile/edge devices due to limited resources such as memory capacity and power consumption. Researchers have carefully designed some lightweight networks. Donegan et al. (2021) used a differentiable NAS method to find efficient CNNs for the Intel Movidius Vision Processing Unit (VPU), achieving state-of-the-art accuracy on ImageNet. An FPGA-based CNN accelerator (field programmable gate array) was proposed (Fan et al., 2020) with an accurate performance model of hardware design. Intelligent edge-cloud scenarios are expected to meet diverse requirements.

All the above studies do not effectively record evolutionary information, so they cannot guide the whole search process based on experience. In contrast, Bayesian optimization (Frazier, 2018) assumes the search space as a Gaussian distribution, learns experience in search processes, and calculates better parameters iteratively (Wistuba; Gupta et al., 2017; Ji et al., 2019; Basha et al., 2021). However, these methods still cost enormous computing resources. As a result, this paper suggests a heuristic target-dependent method that only needs small samples and is entirely automatic.

### Proposed method

From the above, we know that neural architecture search is time-consuming and requires many resources. Therefore, we intend to optimize the hyperparameter exploration process. In short, the main contributions of this paper are as follows:

- Method: We proposed a target-dependent search method that only needs small samples. Besides accuracy, we use precision and recall to promote generalization. Also, our method can find searches that are not working well and stop them early to save time and resources.
- Dataset: We collected eight kinds of rapeseed images and created the dataset RSDS.
- Comparison: Horizontally, we compare each TD-NAS based on VGGNets. Vertically, we explore the TD-NAS based on VGGNet-D (VGGNet-16). Furthermore, we test our method on two additional open datasets, Pl@ntNet and ICL-Leaf.

### Infrastructure and hyperparameters

**Figure 1:**
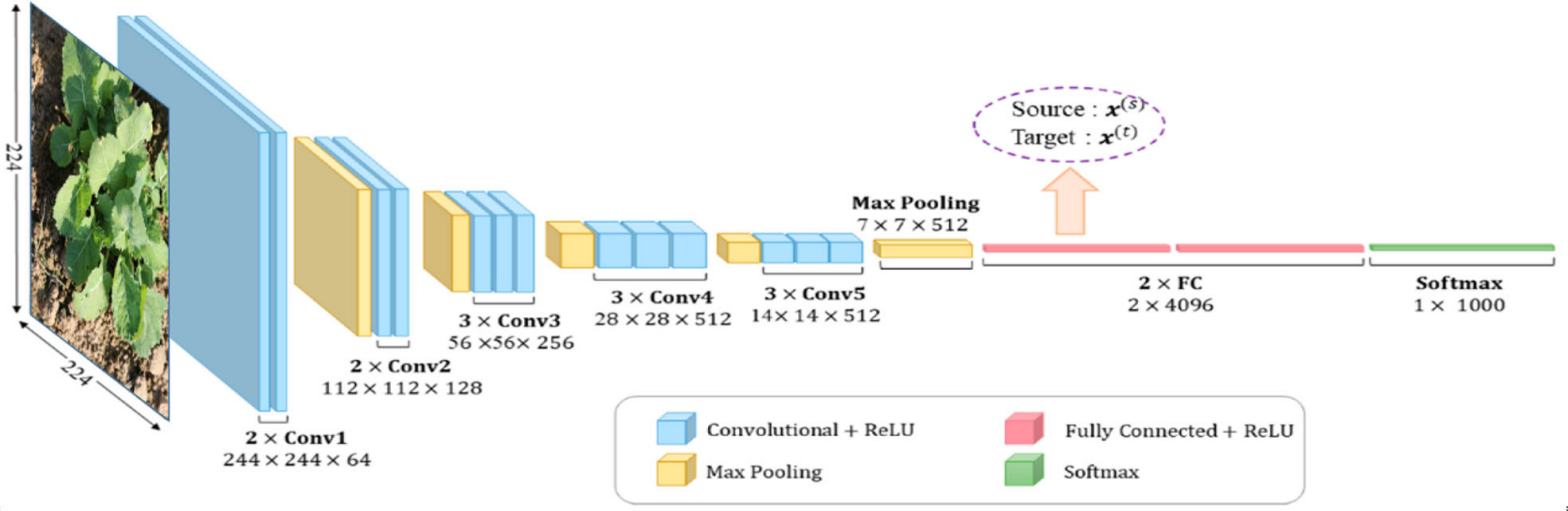
VGGNet-16.

As for the primary network architecture, we chose VGGNet, a typical convolutional network with six deepening structures labeled A, A-LRN, B, C, D, and E. Here we select A, B, D, and E for experiments, and D is the most famous model named VGGNet-16 (**Figure 1**).

We keep the number and position of convolution and pooling layers fixed, while the layer number, the dropout rate, and the neuron number of fully connected layers can be changed. **Table 1** shows the hyperparameter space. It is worth noting that there is at least one fully connected layer, and the number of neurons in the last fully connected layer is eight to produce the output—eight classes of rapeseed.

**Table 1:** Hyperparameter space.

Level	Layer	Name	Values	Type
Architectural	*FC*	*LN*	{1, 2, 3}	Integer
Internal	*FC*	*DR*	{0.0, 0.1, 0.2, 0.3, 0.4, 0.5, 0.6, 0.7, 0.8, 0.9}	Float
Internal	*FC*	*NN*	{512, 1,024, 2,048, 4,096}	Integer
External	*OU*	ω_2 =_ 1- ω_1_	{1.0, 0.9, 0.8, 0.7, 0.6, 0.5}	Float
External	*OU*	β	{0.5, 1.0, 2.0}	Float

FC, fully connected; LN, layer number; DR, dropout rate; NN, neuron number; OU, output. ω, β: parameters in formula (2) (discussed in the next section.)

### Search principle and Bayesian optimization

The aim is to find the hyperparameters of a model with the best performance on verification sets. Let *T* be the objective function for getting maximum accuracy (*ACC*).


(1) $$hp^{*}=\underset{hp\in D}{\text{argmax}}T\left(hp\right)$$

In formula (1), *D* is a hyperparameter space. We can create a model for each *hp* in *D*, train the model, and evaluate its performance on verification sets. This paper separates the RSDS dataset into three parts: the training set, the validation set, and the test set, with a ratio of 7:2:1. We use formula (2) as the judgment criteria (*jc*) for evaluating model qualities:


(2) $$jc=\omega_{1}Acc+\omega_{2}F_{\beta }$$

Here, *F_β_* (balanced *F*-score) is the harmonic average of precision and recall. Usually, the smaller the F-score, the better the generalization. Therefore, we refine formula (1) to formula (3):


(3) $$hp^{*}=\underset{hp\in D}{\text{argmax}}T\left(hp,\;\;jc\right)$$

The following **Algorithm 1** gives the naive hyperparameter search process:

Algorithm 1 HPS: Hyperparameters Search.
**Input:** Space_D, Data_T, Data_VOutput: hp* (1) Get the training set (Data_T) and the verification set (Data_V). (2) Select a judgment criterion: *ACC.* (3) Set hyperparameter search space (Space_D) and initialize one hyperparameter (hp): (4) Generate a CNN model with the hp. (5) Train the model, and verify it. (6) Select the next hyperparameter (hp), repeat (4), or Quit. (7) Output the optimal hyperparameter (hp*).

**Algorithm 1** does not record evolutionary information, so it cannot guide search processes effectively. Even if adopting a random search, uncertainty still exists.

We propose a heuristic target-dependent search method. The so-called heuristic means our approach can evaluate a better location and start a new search. Here, we choose Bayesian optimization, which assumes the superparameter space as a Gaussian distribution and obtains better candidates each time. Our method introduces a stop criterion to reduce the search scale without lowering generalization. The so-called target-dependent means that the explored architecture is not universal and only valid for specific crops. We can quickly rerun the proposed method to search out new architectures when facing new species.

Bayesian optimization has two components: (a) Bayesian statistics for constructing an objective function (typically a Gaussian process); and (b) acquisition function for calculating the following sampling points. After initializing several points, Bayesian optimization can calculate *a posteriori* and iterate reasoning until meeting an exit condition. Algorithm 2 shows our TD-NAS method based on Bayesian optimization, where GP is a Gaussian process, Acq_F is an acquisition function, and Dyn_QF is a dynamic quit function. In Algorithm 2, we focus on steps (6), (4), and (3). Step (4) costs massive resources for VGGNet training and verifying, so step (6) should select hyperparameters elegantly to reduce the number of models. Furthermore, step (4) should stop the training and verifying processes when there are unpromising detections. Step (3) checks the dynamic quit conditions and decides whether to quit or not.

Algorithm 2 TD-NAS: Target-dependent neural architecture search.
**Input:** VGGNet, Space_D, Data_T, Data_V, GP, Acq_F, Dyn_QFOutput: x* (1) Init S = {(x_i_, y_i_)}, y_i_ = f(x_i_), x_i_ ϵ *Space_D*, let f~GP(μ, K). (2) Select a judgment criterion: *jc*. (3) While not Dyn_QF() do: (4) Train and verify VGGNet(x, Data_T, Data_V) with unpromising detections. (5) Calculate p(y | x, S). (6) Acq_F(x, p(y | x, S)), get x_new_. (7) y_new_ = f(x_new_). (8) S = S U (x_new_, y_new_). (9) Output the optimal hyperparameter (x*).

Step (6): The acquisition function strikes a balance between exploration and exploitation.

In Bayesian optimization, the acquisition function (Acq_F) is critical for generating points according to prior knowledge. Exploitation means evaluating at expected points because global optima are likely to reside there. Exploration means considering uncertain points is helpful because objects tend to be far from where we have measured them. Usually, there are three typical acquisition functions: expected improvement (EI), entropy search (ES), and knowledge gradient (KG). The expected value of EI is easy to figure out, which makes it a popular acquisition function.

Let $f_{n}^{*}={\text{max}}_{m\leq n}f\left(x_{m}\right)$ be the max previous value. We have one other position, x, to be evaluated, and then we get f (x). Now, the best-observed point is either *f(x)* or $f_{n}^{*}$ . The improvement is then $f\left(x\right)-f_{n}^{*}$ ; if this quantity is positive, else 0, mark as ${\left[\text{f}\left(\text{x}\right)-f_{n}^{*}\right]}^{+}$ for convenience. Unfortunately, we should train and validate the entire network to get *f(x)*. Instead, we can take the expected value of this improvement and define formula (4):


(4) $$x_{n+1}={\text{argmaxEI}}_{n}\left(x\right)$$

Here, ${\text{EI}}_{n}\left(x\right)=E_{n}\left[{\left[\text{f}\left(\text{x}\right)-f_{n}^{*}\right]}^{+}\right]$ , and *E_n_* indicates that the expectation is taken under the posterior distribution, as shown in formula (5): *f(x)* given *x*_1:_ *_n_*, *y*_1:_ *_n_* is normally distributed with mean *μ_n_* (x) and variance $\sigma_{n}^{2}\left(\text{x}\right)$ .


(5) $$f\left(x\right)|f\left(x_{1:n}\right)\sim \text{Normal}\left(\mu_{n}\left(x\right),\sigma_{n}^{2}\left(x\right)\right)$$

Unlike the *f(x)* in step 4 of Algorithm 2, EI*_n_* (*x*) is low cost to observe and allows for easy evaluation of first- and second-order derivatives, as shown in formula (6):


(6) $${\text{EI}}_{n}\left(x\right)=\Delta_{n}\left(\text{x}\right)\Phi \left(\frac{\Delta_{n}\left(\text{x}\right)}{\sigma_{n}\left(\text{x}\right)}\right)+\sigma_{n}\left(\text{x}\right)\varphi \left(\frac{\Delta_{n}\left(\text{x}\right)}{\sigma_{n}\left(\text{x}\right)}\right)$$

The definitions of *Φ*(),*φ*() can be found in (Jones et al., 1998). Here, $\Delta_{n}\left(\text{x}\right)=\mu_{n}\left(\text{x}\right)-f_{n}^{*}$ is the expected difference between the proposed point *x* and the previous best. Note that EI*_n_* (*x*) balances between high expected quality (Δ*_n_* (*x*)) versus high uncertainty (σ*_n_* (*x*)).

**Step (4):** Training and validating the VGGNet without unpromising detections.

**Figure 2:**
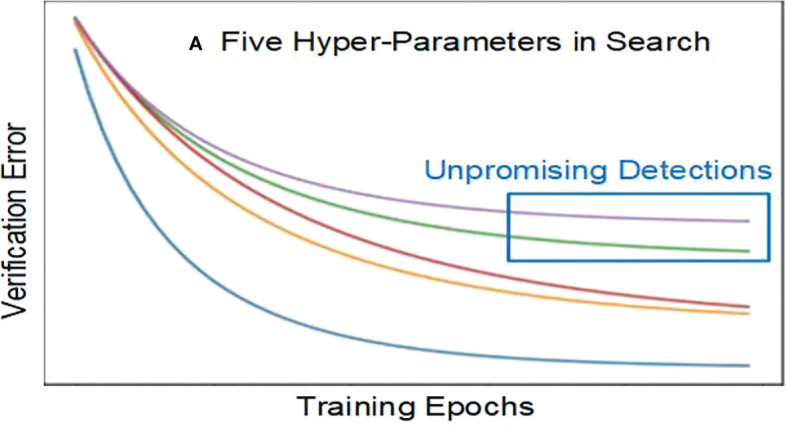
Unpromising detections.

We calculate verification errors when training and verifying. If the error exceeds the average prior value, it is unpromising to go further. **Figure 2** shows five hyperparameters in search, including two unpromising detections. Stopping these unpromising detections early can save resources and time.

**Step (3):** Making dynamic quit decisions.

**Figure 3:**
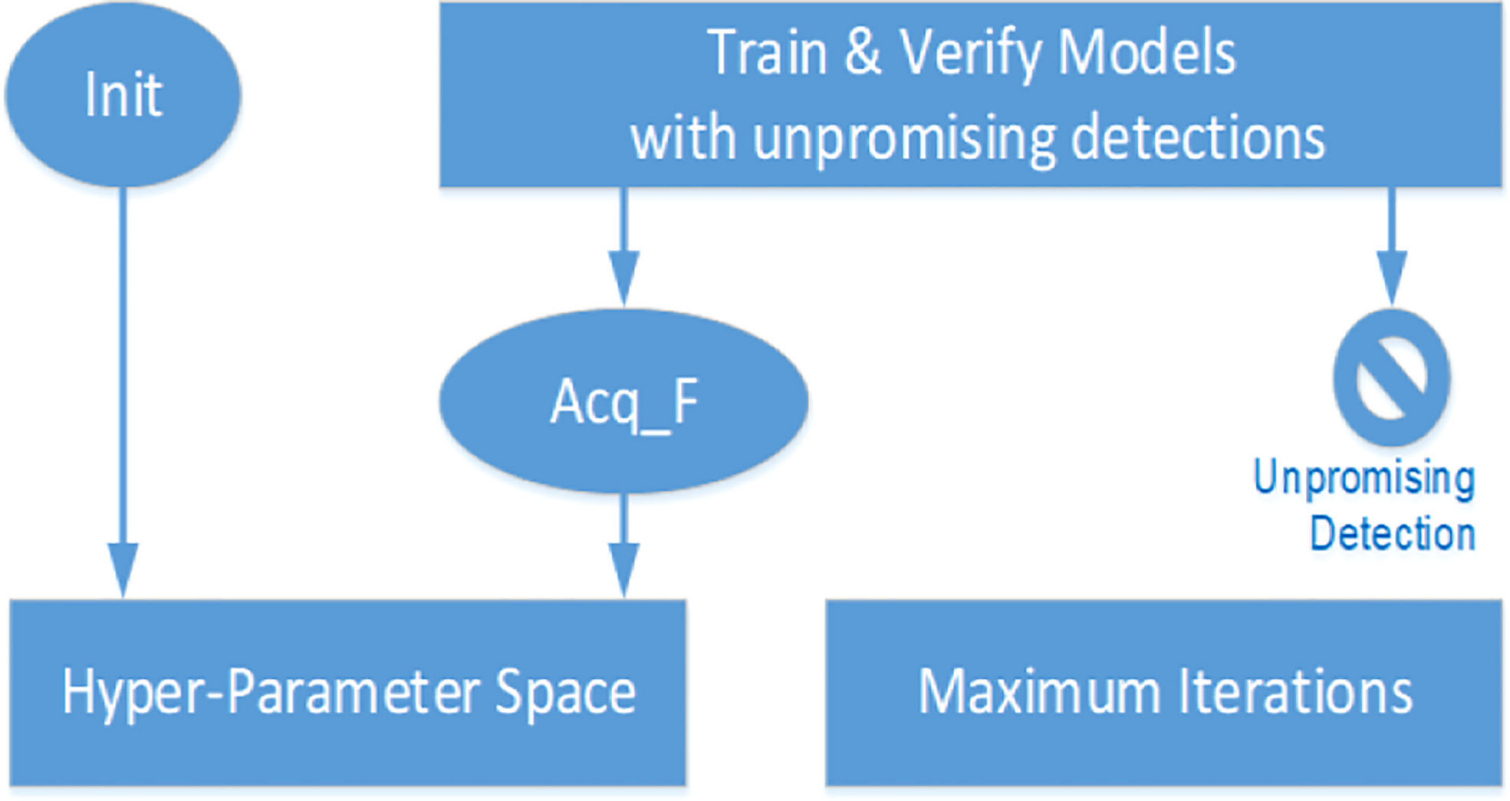
Dynamic quit conditions. Generation approach.

**Figure 3** gives dynamic quit conditions and their generation approach. To control the search process, a dynamic quit function (Dyn_QF) uses these conditions, including whether the queue of the hyperparameter space is empty or the maximum number of iterations has been reached.

## Experiment and discussion

### Dataset and experimental condition

We took photos using a Canon EoS6D camera, which has 20.2 million effective pixels and a maximum resolution of 5,472 × 3,648. We then resized each image to 224 × 224 pixels to improve the processing speed. To run programs, we used an HP-OMEN laptop with an i9-9880H CPU, 32 GB of memory, an NVIDIA RTX2080 graphics card (8 GB), and Python installed.

**Figure 4:**
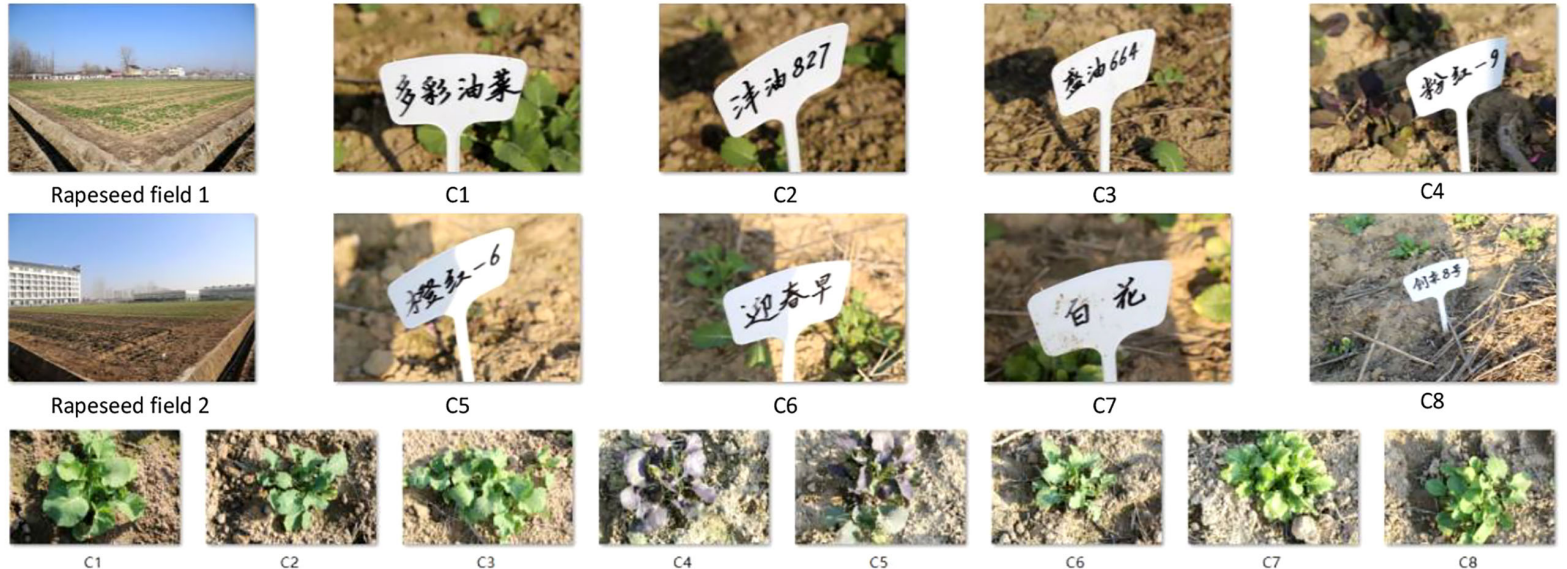
Rapeseed dataset (RSDS).

We collected rapeseed images at an experimental station of Anhui Agricultural University, located at 117.2° east longitude and 31.5° north latitude, in Sanhe town, Hefei, China. We obtained eight kinds of rapeseed images, at least 1,000 of each class (*C_i_* |*i* = 1,…, 8), as shown in **Figure 4**, named RSDS. We divided the RSDS into three parts: training set (Tr), verification set (V), and test set (Te), with a ratio of 7:2:1. We randomly obtained *RSDS-0.1K* with (Tr, V, Te = 700, 200, 100) images per class. Using data augmentation, we obtained more sets as follows: *RSDS-0.2K* (Tr, V, Te = 1,400, 400, 200), *RSDS-0.4K* (Tr, V, Te = 2,800, 800, 400), and *RSDS-1.0K* (Tr, V, Te = 7,000, 2,000, 1,000).

### Result discussion

We use VGGNet as the base framework. When training, the initial learning rate is 0.01, and the epoch size is 50. For comparing two algorithms, “better” means (a) fewer attempts for the same score and (b) a higher score after the same number of tries.

For horizontal comparisons, (a) we set *ω*_1_ = 1, which means only accuracy is the evaluation indicator. In **Table 2**, the verification accuracy of TD-NAS based on VGGNet-16 reaches 81.38%. However, the result obtained on VGGNet-E (VGGNet-19) is worse than the original, indicating that Bayesian optimization also has limitations in dealing with deep networks. The number of neurons in the last fully connected layer fixes eight to output the probability values of rapeseed classes through a Softmax function. (b) Do not fix ω_1_. Instead, use *jc* as the indicator in formula (2) (**Table 3**).

**Table 2:** TD-NAS and original VGGNet (A, B, D, and E) (*RSDS-0.1K*, *ω*_1_ = 1).

	VGGNet-A		VGGNet-B		VGGNet-D		VGGNet-E	
	Original	TD-NAS	Original	TD-NAS	Original	TD-NAS	Original	TD-NAS
*V-ACC*	74.06%	78.81%	77.38%	77.94%	79.19%	**81.38%**	80.75%	78.44%
*FC*								
*LN*	3	3	3	3	3	3	3	3
*DR*	{0.7, 0.7, 0.0}	{0.6, 0.4, 0.0}	{0.7, 0.7, 0.0}	{0.7, 0.5, 0.0}	{0.7, 0.7, 0.0}	{0.6, 0.5, 0.0}	{0.7, 0.7, 0.0}	{0.5, 0.3, 0.0}
*NN*	{4,096, 4,096, 8}	{2,048, 2,048, 8}	{4,096, 4,096, 8}	{2,048, 2,048, 8}	{4,096, 4,096, 8}	{4,096, 2,048, 8}	{4,096, 4,096, 8}	{2,048, 2,048, 8}
*OU*								
*ω*_1_	1	1	1	1	1	1	1	1
*β*	–	–	–	–	–	–	–	–

V-ACC, verification accuracy; FC, fully connected; LN, layer number; DR, dropout rate; NN, neuron number; OU, output. Bold means To highlight the biggest verification accuracy (V-ACC).

Bold values mean to highlight the biggest verification accuracy (V-ACC).

**Table 3:** TD-NAS (*RSDS-0.1K*, *ω*_1_ not fixed).

	TD-NAS			
	VGGNet-A	VGGNet-B	VGGNet-D	VGGNet-E
*V-ACC*	77.56%	75.13%	**81.06%**	79.94%
*FC*				
*LN*	3	3	3	3
*DR*	{0.6, 0.4, 0.0}	{0.7, 0.5, 0.0}	{0.6, 0.5, 0.0}	{0.5, 0.3, 0.0}
*NN*	{2,048, 2,048, 8}	{2,048, 2,048, 8}	{4,096, 2,048, 8}	{2,048, 2,048, 8}
*OU*				
ω_1_	0.9	0.9	0.8	0.9
β	2	0.5	1	0.5

V-ACC, verification accuracy; FC, fully connected; LN, layer number; DR, dropout rate; NN, neuron number; OU, output. Bold means To highlight the biggest verification accuracy (V-ACC).

Bold values mean to highlight the biggest verification accuracy (V-ACC).

Keep the fully connected parameters from **Table 2** unchanged and take into account *ω*_1_ and *β*. After searching, we still get the highest verification accuracy (81.06%, **Table 3**) based on VGGNet-D, which is slightly lower than the accuracy of VGGNet-16 (81.38%, **Table 2**). However, the verification accuracy of TD-NAS based on VGGNet-E has increased from 78.44% (**Table 2**) to 79.94% (**Table 3**).

Now, for vertical comparisons, (a) we use four datasets (**Table 4**) to search for TD-NAS on VGGNet-D (VGGNet-16). (b) We choose the model made by *RSDS-0.1K*, figure out how accurate it is on all of the test sets, and compare it to other models (**Table 5**). In **Table 4**, it can be seen that the bigger the dataset size, the higher the verification accuracy (91.23%, **Table 4**). It is worth noting that from *RSDS-0.4K* to *RSDS-1.0K*, the promotion of verification accuracy is only 1.95%, but the amount of training data has increased by 1.5 times. Meanwhile, the time consumptions of *RSDS-0.1K*, *RSDS-0.2K*, *RSDS-0.4K*, and *RSDS-1.0K* are approximately 00:42:32, 01:11:36, 04:20:38, and 07:26:00, respectively. The costs of training and verification vary greatly, but the benefits are limited.

**Table 4:** TD-NAS on VGGNet-D (VGGNet-16) (*ω_1_ not fixed*).

	TD-NAS on VGGNet-D (VGGNet-16)			
	*RSDS-0.1K*	*RSDS-0.2K*	*RSDS-0.4K*	*RSDS-1.0K*
*V-ACC*	81.06%	86.53%	89.28%	**91.23%**
*FC*				
*LN*	3	3	3	3
*DR*	{0.6, 0.5, 0.0}	{0.7, 0.3, 0.0}	{0.4, 0.6, 0.0}	{0.5, 0.4, 0.0}
*NN*	{4,096, 2,048, 8}	{4,096, 2,048, 8}	{2,048, 2,048, 8}	{2,048, 1,024, 8}
*OU*				
ω_1_	0.8	0.9	0.9	0.8
β	1	0.5	1	2

V-ACC, verification accuracy; FC, fully connected; LN, layer number; DR, dropout rate; NN, neuron number; OU, output. Bold means To highlight the biggest verification accuracy (V-ACC).

Bold values mean to highlight the biggest verification accuracy (V-ACC).

**Table 5:** Test accuracy comparison.

T-ACC	TD-NAS on VGGNet-D (VGGNet-16)			
	RSDS-0.1K	RSDS-0.2K	RSDS-0.4K	RSDS-1.0K
RSDS-0.1K	81.13%	–	–	–
RSDS-0.2K	79.94%	81.44%	–	–
RSDS-0.4K	80.22%	–	83.13%	–
RSDS-1.0K	81.68%	–	–	82.73%

T-ACC, test accuracy.

The TD-NAS mentioned above on VGGNet-D (VGGNet-16) searches each dataset to generate models. **Table 5** shows the accuracy of these models on test sets; the diagonal part of the table shows the accuracy of each model on its own test set, whereas the first column is the accuracy of the model generated by *RSDS-0.1K* on all test sets. It is worth noting that the test accuracy of the model trained on small samples is not much different from that trained on large ones.

**Figure 5:**
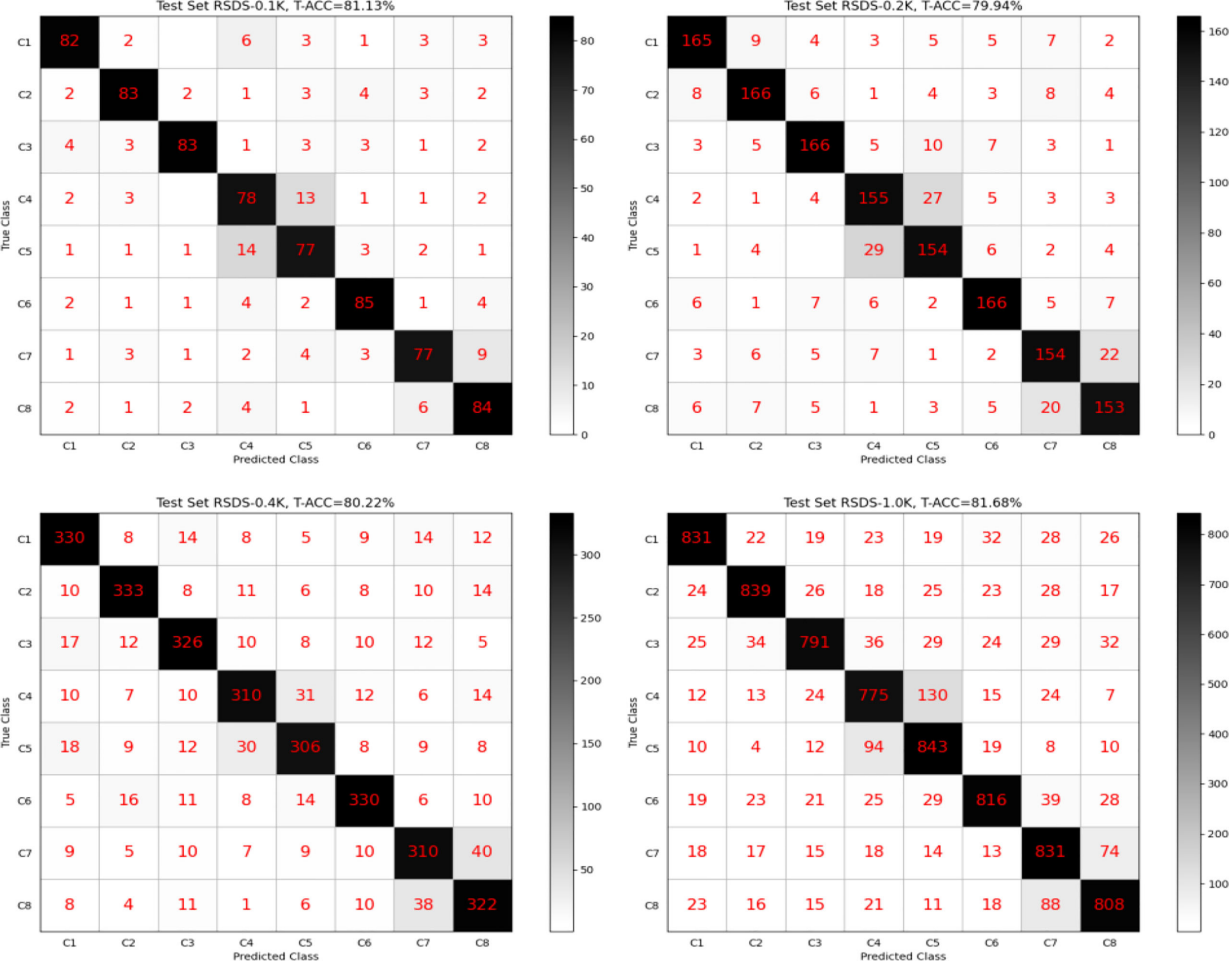
Confusion matrixes generated by TD-NAS on VGGNet-D (VGGNet-16).

**Figure 5** shows four confusion matrixes generated on each test set separately, and the model is TD-NAS on VGGNet-D (VGGNet-16). Overall, our model performed well across different test sets. It is worth noting that the two pairs [C4, C5] and [C7, C8] are far more likely than others to misjudge each other. When a person looks at the rapeseed images of these two pairs, they look very similar in color and shape.

### Pl@ntNet-300K and ICL-Leaf datasets

**Figure 6:**
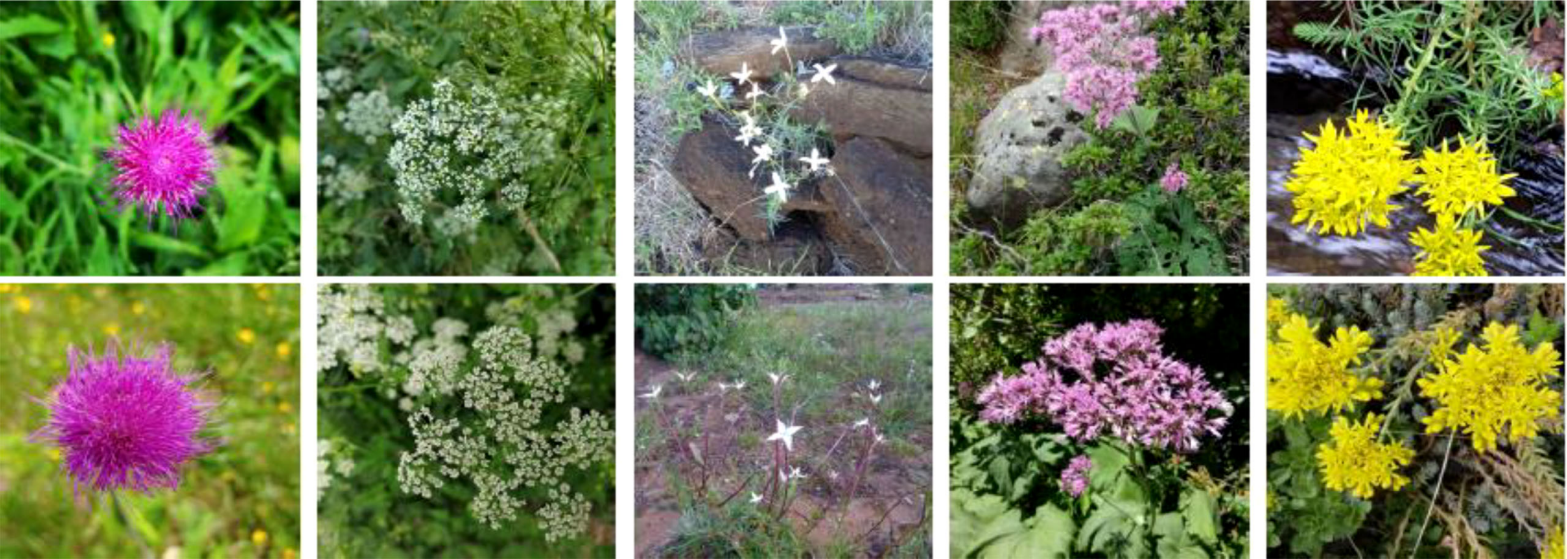
Examples of visually similar images belonging to two different classes.

A novel image dataset with high intrinsic ambiguity was presented (Camille et al., 2021), built explicitly for evaluating and comparing set-valued classifiers. It consists of 306,146 images covering 1,081 species, with two particular features: (a) The dataset has a strong class imbalance, which means that a few species account for most images. (b) Many species are visually similar (**Figure 6**), making identification difficult even for eye experts.

**Table 6** shows that test accuracy depends strongly on the number of images per class. We selected eight species, including *Cirsium arvense*, and searched for architecture based on VGG-16. Let [s1, s2] be the interval of the image number per class (for example (Rahaman et al., 2019; Sun et al., 2019),), and we searched twice with s1 and s2 per class (for >2,000, set [2,500, 3,000]), then calculated the mean accuracy. From **Table 6**, we found that our method got higher accuracy than Ref (Camille et al., 2021). claimed, except for the situation “>2,000”. The results in Ref (Camille et al., 2021). were made with ResNet50, which has 49 convolutional layers and one fully connected layer. This architecture is deeper than ours and better at handling large samples.

**Table 6:** Test accuracy comparison.

Number of images per class	Mean accuracy claimed in (Camille et al., 2021)	Ours, TD-NAS on VGGNet-D (VGGNet-16)
10–50	35%	41.25%
50–500	59%	63.38%
500–2,000	79%	80.97%
>2,000	93%	86.75%

**Figure 7:**
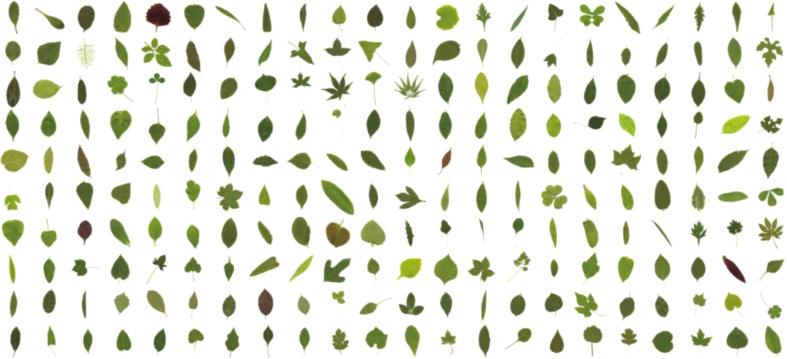
Typical samples for each species.

Another public leaf dataset called the ICL (Hu et al., 2012) was built by the Intelligent Computing Laboratory (ICL) at the Institute of Intelligent Machines, Chinese Academy of Sciences. It contains 16,851 samples from 220 species, with 26 to 1,078 samples per species (**Figure 7**).

We selected eight species, including *Amorpha fruticosa*, and searched for architecture based on VGG-16 two times. We got an average test accuracy of 92.31%, a little more than 92.08% in Ref (Xiao et al., 2010)., which used a traditional machine-learning method named HOG-MMC (orientation histogram based on dimension reduction of maximum edge criterion).

From the above, we proved that our method could quickly discover new architectures when faced with Pl@ntNet-300K and ICL-Leaf datasets. The contribution of our method includes three features: (a) small sample sizes, (b) stable generalization, and (c) free of unpromising detections. In experiments of Pl@ntNet-300K and ICL-Leaf, as for feature a, we selected [500, 2,000] and [1,000, 1,200] images per class and got average test accuracies represented above. As for feature b, we increased the number of images per class to [2,500, 3,000] and [1,500, 2,000], and test accuracy had risen a little (< 6%), but time cost ascended (> 26%). As for feature c, we set a switch to control NAS with or without unpromising detections, and statistics showed that more than 19% of detections are unpromising. To sum up, due to features a and c, we can quickly find out new architectures when faced with new species but still get feature b’s stable generalization.

## Conclusion

This paper aims to design a novel target-dependent neural architecture search method based on VGGNet. This goal was successfully achieved on a self-built dataset with eight kinds of rapeseed images. We select accuracy, precision, and recall as the evaluation indicators. We adopt Bayesian optimization to obtain better candidate parameters and introduce a stop criterion for optimizing the dynamic search process. Results show that the test accuracy of the model trained on small samples is not much different from those trained on large ones. The generalization of the model generated by our method is not sensitive to dataset size, making it meaningful to search out models from small samples when facing new objects. For example, we tested our method on two other open datasets, Pl@ntNet and ICL-Leaf.

**Figure 8:**
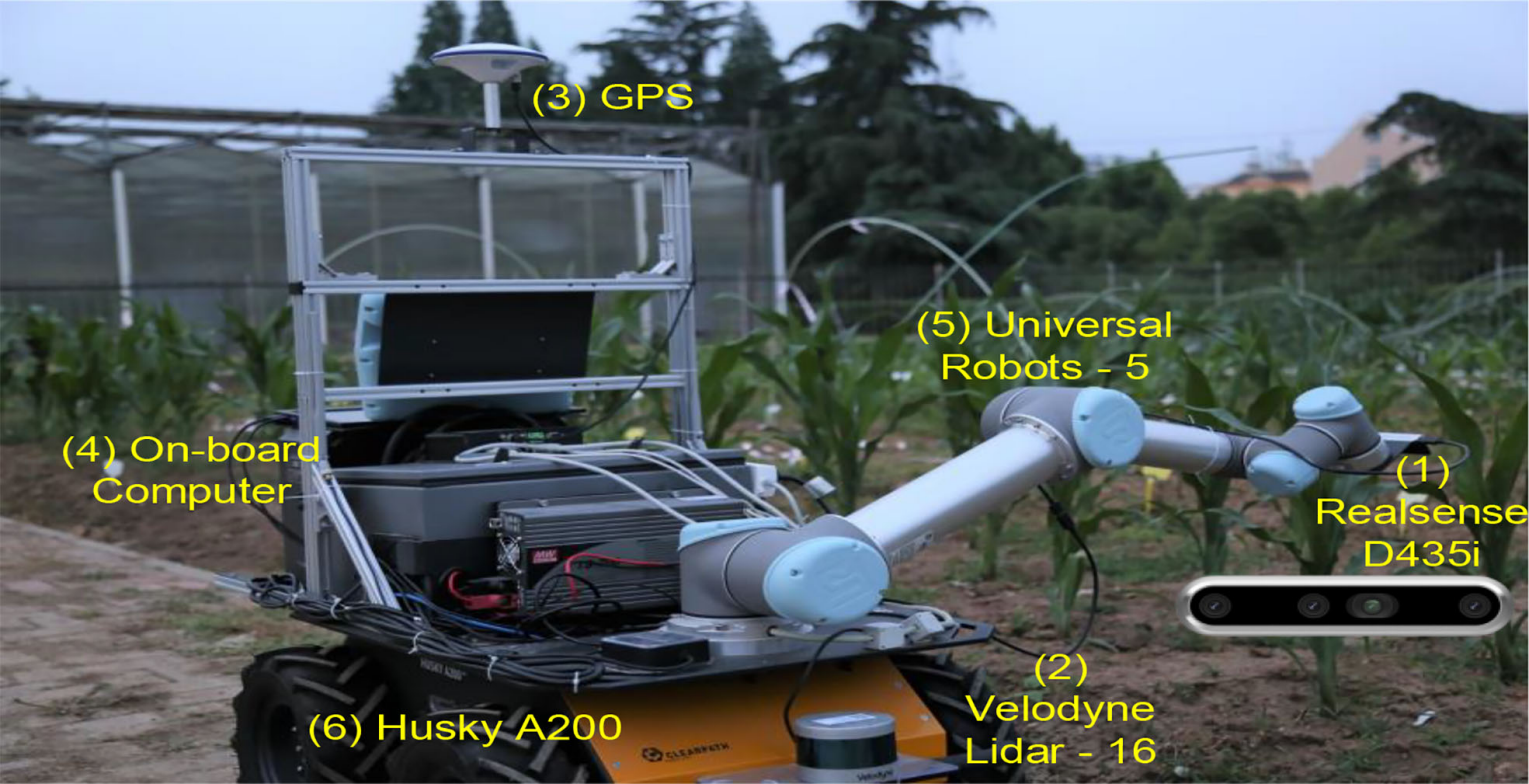
Our robot platform (capture pictures by (1) and run models on (4)).

Due to the enormous model structure and parameter space, this paper only dealt with fully connected layers and the output layer. We kept the original network structure unchanged, such as the kernel and pooling size in convolutional layers. However, the full network structure search, including convolutional layers, is universal without margin. Some literature has pointed out that Bayesian optimization is only suitable for medium-sized problems (Shahriari et al., 2015). When faced with new objects, how to quickly search a minimized network structure is still an attractive topic. **Figure 8** shows our robot platform, with our models running on (4), an onboard computer, and real-time images captured by (1), a Realsense D435i camera. We continue to explore in-depth research questions, such as changing kernels and pooling sizes in convolutional layers (Franchini et al., 2022). We are thinking about using the proposed method on more CNN frameworks in the future.

## Data availability statement

The self-build dataset RSDS will be made available by the authors. Another two open datasets (Pl@ntNet and ICL-Leaf) can be found on the official sites or the Internet, such as: https://zenodo.org/record/5645731.

## Author contributions

LYF and YR planned and designed the research. JXL, JT, and QLH performed the experiments. LYF wrote the manuscript. SWL supervised this work and reviewed the manuscript. All authors contributed to the article and approved the submitted version.

## Funding

948 Project of Introduction of International Advanced Agricultural Science and Technology by Ministry of Agriculture, Ministry of Agriculture, China, No.2016-X34.

## Conflict of interest

The authors declare that the research was conducted in the absence of any commercial or financial relationships that could be construed as a potential conflict of interest.

## Publisher’s note

All claims expressed in this article are solely those of the authors and do not necessarily represent those of their affiliated organizations, or those of the publisher, the editors and the reviewers. Any product that may be evaluated in this article, or claim that may be made by its manufacturer, is not guaranteed or endorsed by the publisher.
